# Supplementary material for: Phototransduction in Anuran Green Rods: Origins of Extra-Sensitivity
Source: Int J Mol Sci. 2021 Dec 13;22(24):13400. doi: 10.3390/ijms222413400 (PMC8707487; doi:10.3390/ijms222413400)
Supplement: Supplementary file 1 [file ijms-22-13400-s001.zip › Figure S1.pdf]

Supplementary material for article:
"Phototransduction in anuran green rods: origins of extra-sensitivity"

Luba A. Astakhova[1], Artem D. Novoselov[1], Maria E. Ermolaeva[1], Michael L. Firsov[1], Alexander Yu. Rotov[1,] *

[1]Sechenov Institute of Evolutionary Physiology and Biochemistry, 44 Torez Prospect, 194223 St. Petersburg, Russia; office@iephb.ru

*Correspondence: rotovau@gmail.com

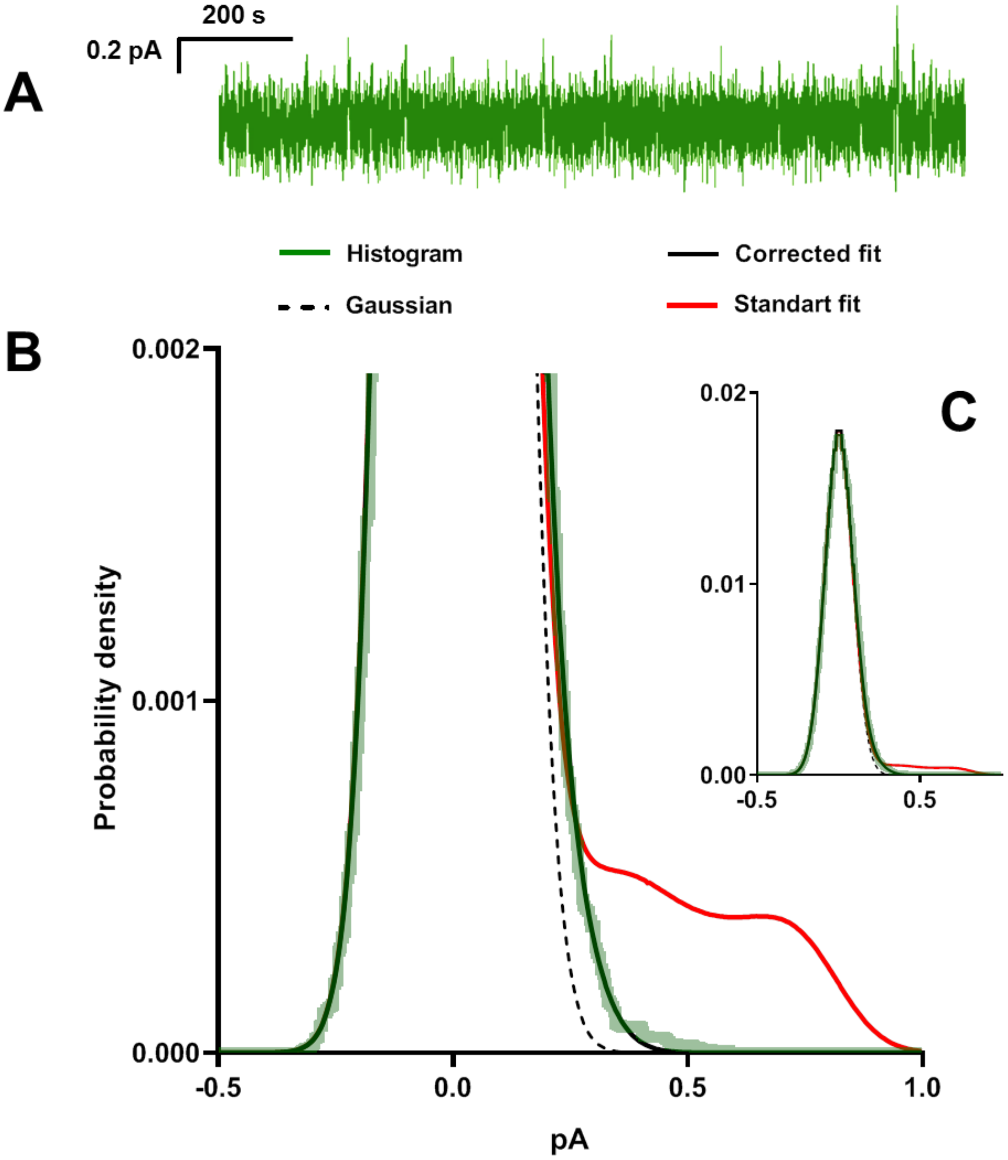

Figure S1. Example of overestimation of green rod dark event amplitude from Poisson analysis. (**A**) Dark current record of toad green rod. (**B, C**) Corresponding probability density histogram (green line) with asymmetric shoulder representing the dark noise. Dashed line is the best fit for histogram of dark record with excised discrete events by Gaussian function (σ =0.088). Black curve is the best fit by the function from eq. 3 (see main text) that contain a correcting coefficient for Poisson-calculated single photon response and it gives the frequency of dark events 0.015 s^{-1}. Red curve represents virtually the the same function (with the same dark event frequency) but without response amplitude correction. To obtain any acceptable fit without such correction one should set the frequency of discrete events as extremely low (less than one event per record duration) which is in conflict with the result of direct analysis of the dark current record (**A**) containing clearly visible discrete events.
